# Current Practice of Therapeutic Mammaplasty: A Survey of Oncoplastic Breast Surgeons in England

**DOI:** 10.1155/2016/1947876

**Published:** 2016-03-23

**Authors:** Shweta Aggarwal, Sekhar Marla, Donald Nyanhongo, Sita Kotecha, Narendra Nath Basu

**Affiliations:** Queen Elizabeth Hospital NHS Foundation Trust, Birmingham B15 2TH, UK

## Abstract

*Introduction*. Therapeutic mammaplasty (TM) is a useful technique in the armamentarium of the oncoplastic breast surgeon (OBS). There is limited guidance on patient selection, technique, coding, and management of involved margins. The practices of OBS in England remain unknown.* Methods*. Questionnaires were sent to all OBS involved with the Training Interface Group. We assessed the number of TM cases performed per surgeon, criteria for patient selection, pedicle preference, contralateral symmetrisation, use of routine preoperative MRI, management of involved margins, and clinical coding.* Results*. We had an overall response rate of 43%. The most common skin resection technique utilised was wise pattern followed by vertical scar. Superior-medial pedicle was preferred by the majority of surgeons (62%) followed by inferior pedicle (34%). Twenty percent of surgeons would always proceed to a mastectomy following an involved margin, whereas the majority would offer reexcision based on several parameters. The main absolute contraindication to TM was tumour to breast ratio >50%. One in five surgeons would not perform TM in smokers and patients with multifocal disease.* Discussion*. There is a wide variation in the practice of TM amongst OBS. Further research and guidance would be useful to standardise practice, particularly management of involved margins and coding for optimal reimbursement.

## 1. Introduction

Therapeutic mammaplasty (TM) is a well-established oncoplastic procedure [1, 2]. It offers breast conservation, particularly with larger tumours, by using the principles of a reduction mammaplasty and mastopexy to reshape the breast. In addition, it is likely to positively affect the quality of life as seen with breast conservation [3]. In an era of improving systemic treatments, particularly the use of neoadjuvant treatment to downsize tumours, TM is an ideal option in carefully selected patients.

With the advent of oncoplastic breast surgery, there is limited data on the practices of surgeons throughout the UK. Despite a few institutional reports, no national database on practices or guidelines exists [4–6]. Selection criterion, operative techniques, management of involved margins, and training of surgeons performing this procedure are unknown with possible varying practices between units.

The United Kingdom Training Interface Group (TIG) pioneered the first structured training programme in oncoplastic breast surgery in 2002. These fellowships at various recognised oncoplastic centres offer recognised training to breast and plastic surgical trainees interested in offering advanced oncoplastic procedures. A database of surgeons involved with the TIG has been maintained for over a decade.

There are several key steps when planning a TM. Appreciation of tumour biology with attention to tumour size to breast ratio needs to be considered when assessing suitability. Patients with comorbidities may not be selected for this procedure as postoperative complications may impact on adjuvant treatments [5]. At the time of surgery, careful consideration should be given to the type of dermoglandular pedicle (primary or secondary) used to fill any defect to ensure proper remodeling of the breast. Healthcare economics and operative logistics may dictate whether a simultaneous symmetrisation procedure is offered. This may increase the operative time or involve a second procedure. At present, there is limited guidance on reimbursement or clinical coding for therapeutic mammaplasty as it is essentially a hybrid of an oncological resection and a plastic surgical procedure of a reduction mammaplasty/mastopexy.

Preoperative localisation and intraoperative imaging of the tumours are critical steps to maximise the chances of complete resection margins. Frozen section is not standard practice in the UK. There is no consensus or any available data on management of patients with involved resection margins following a therapeutic mammaplasty. This is despite a recent meta-analysis assessing optimal margin within breast conserving surgery [7].

We report the results of the first national survey of oncoplastic breast surgeons involved with the TIG and assess their practices of TM. In particular, we ascertained what selection criteria surgeons used when considering TM, which of the various described techniques were used or preferred, and how involved surgical margins were managed.

## 2. Methods

The TIG database was accessed to obtain the list of all oncoplastic trainees and trainers affiliated with the TIG National Oncoplastic Fellowship. The format of the questions included open- and close-ended statements, Likert scale, and yes/no responses. Internal validation was included in the design by the collection of questionnaire data from 2 other breast units. The questionnaire was first piloted amongst local surgeons for ease of response, validity, and relevance. Information obtained through the questionnaire included original surgical specialty (breast or plastic surgery) of the respondents; the number of cases they performed annually; preoperative selection criteria; contraindications to surgery; dermoglandular pedicle use; and skin resection technique. Data about whether surgeons offered an immediate symmetrisation procedure and what operating code was used (OPCS code: Office of Population Consensus and Surveys) was also obtained [8]. A reminder questionnaire was sent to nonrespondents after 3 weeks. SurveyMonkey^©^ was used for data collection and Fisher's exact test was performed to check for statistical significance using SPSS^©^ Statistics 22 Software package.

## 3. Results

The TIG database was comprised of 75 previous TIG fellows or trainers. The overall response rate was 43% (*n* = 33). Of these, 91% were breast surgeons and 9% were plastic surgeons. Half the surgeons had performed more than 10 and half less than 10 TMs over the preceding 12 months. Most of the surgeons (60%) had performed between 6–20 TMs. 15% had performed less than 6 and 24% more than 20 TMs.

**Figure 1:**
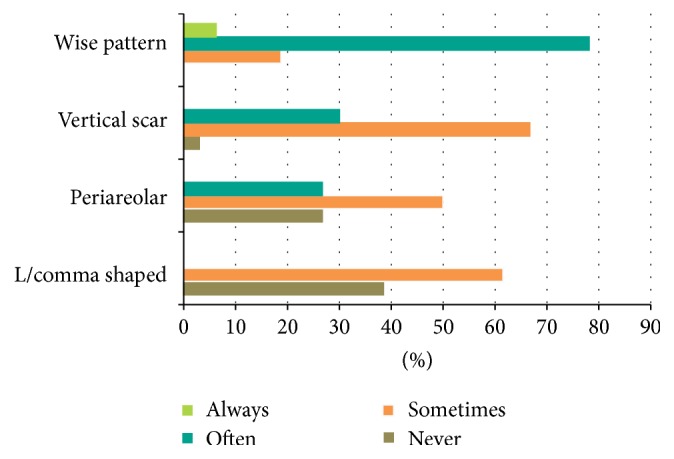
How often do you use the following skin resection techniques in your practice?

Wise pattern was the preferred skin excision technique and 78% of surgeons reported that they use it often followed by vertical scar (30%). Periareolar technique was used less commonly and L/comma shaped only infrequently (Figure 1).

**Figure 2:**
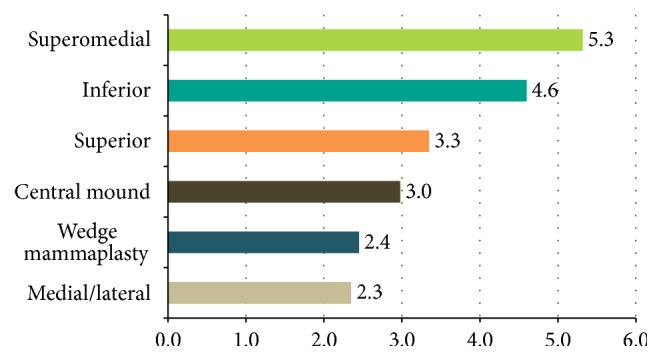
Nipple areolar complex pedicle. Please rank the pedicle most commonly used (1 = most often used, 6 = least often used, average scores out of 6).

62% of respondents reported that they used the superomedial pedicle most often and 34% used inferior pedicle (Figure 2). Only 20% of respondents always offered a contralateral symmetrising procedure at the time of primary surgery, while 13% said that they would never offer this procedure simultaneously. Most surgeons (57%) reported that they would offer this procedure sometimes, for example, if there was a large difference between breast sizes.

Most respondents stated they performed MRI sometimes depending on certain indications. The common indications mentioned were lobular cancer, discrepancy in size on imaging, dense breasts, and neoadjuvant chemotherapy. 15% of respondents said they never perform an MRI and 3% would use it routinely before every procedure. The use of intraoperative imaging was not assessed in the survey.

The procedure was performed exclusively by breast surgeons according to 97% of respondents, while 3% performed it as a joint procedure between breast and plastics.

With regard to coding, 45% of respondents coded the procedure as WLE and reduction mammaplasty, 31% as reduction mammaplasty only, and 5% as wide local excision only.

20% of respondents would always offer mastectomy for involved margins and 3% would always offer reexcision. Subgroup analysis showed that numbers of mammaplasties performed per year did not affect decisions on mastectomy for involved margins (*p* = 0.66). Most surgeons would consider either approach depending on the patient, the pathology of disease, and MDT discussion.

The suitability of the patient for TM was routinely discussed in the oncoplastic MDT by 33% of respondents, while others discuss this at the breast plenary MDT (76%).

**Figure 3:**
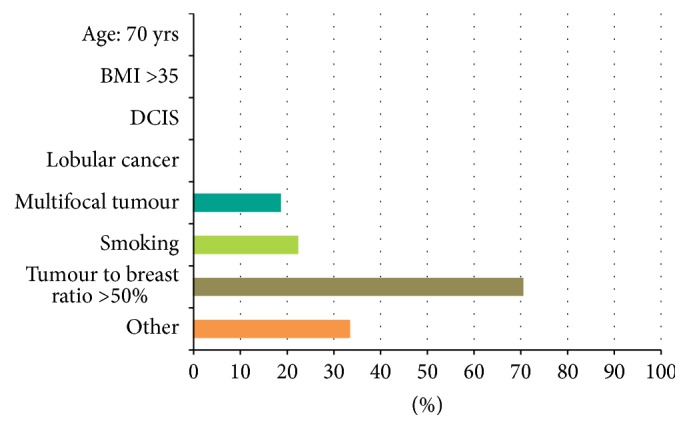
What would you consider as absolute contraindications to performing the procedure?

Most surgeons (70%) would consider a tumour to breast ratio of >50% as an absolute contraindication to performing the procedure. Smoking was considered as an absolute contraindication by 22% of respondents and multicentric tumours were by 18% (Figure 3). Other contraindications mentioned in the comments were previous radiotherapy, locally advanced breast cancer, diabetes, and significant atherosclerotic disease.

## 4. Discussion

In our study group, oncoplastic breast surgeons are most commonly performing TM with only a few units performing joint procedures. In the current survey, there were too few plastic surgeon responses to gauge national practice, which might be a reflection of oncoplastic fellowships historically being undersubscribed by plastic surgeons.

Half of the respondents performed more than 10 and half less than 10 procedures per year. Only 6 (9% of respondents) performed more than 30 TMs per annum. This survey has shown a wide variation in practices amongst surgeons and may reflect the growing evidence base for this procedure. Wise pattern skin resection was the preferred technique by the majority of surgeons. This may reflect surgeons' familiarity with this skin resection technique and that this procedure is preferentially offered to large breasted women requiring skin resection [9]. It also allows a very good access for tumour excision and remodeling of the breast. 30% of respondents did report often using vertical scar and periareolar techniques, suggesting that their role in small breasted women may be expanding. The existence of some degree of ptosis is an important factor in determining the suitability of a patient for TM in small breasted women [6]. The choice of pedicle in TM is largely determined by the location of tumour but may also depend on surgeons' own preference or familiarity with a specific technique.

A constant finding amongst surgeons surveyed in the UK and US was that up to 70% of surgeons use the inferior pedicle as their preferred technique in reduction mammaplasty (noncancer) [9, 10]. In contrast, the most common pedicle used in our cohort was superomedial followed by inferior pedicle which is supported by other studies reporting the same preference [11]. This finding highlights the important differences in planning a TM compared to reduction mammaplasty (noncancer).

The most common site for breast cancer is the upper outer quadrant and either pedicle can be used in this situation. The superomedial pedicle is versatile and can be used for tumour resection in a number of locations. For tumours located in the lower pole of the breast, the tumour resection can be incorporated into the sector of breast tissue removed as part of the mammaplasty. For outer region tumours, the pedicle can be rotated and the defect filled with an extended pedicle or secondary pedicle [6]. The multiple scenarios where this pedicle can be used may account for the observed preference amongst oncoplastic breast surgeons.

The potential benefits of offering a simultaneous contralateral reduction/mastopexy at the time of TM are avoidance of further surgery and achieving symmetry. This is of particular importance in large breasted women undergoing big resections who would otherwise be significantly asymmetrical. Bilateral surgery is resource intensive requiring longer operating times or additional surgeons with several respondents suggesting that funding for contralateral surgery was not always available. An important consideration is the deleterious effects of radiotherapy in terms of shape/size of the index breast resulting in unpredictable asymmetry in the future [12]. This may account for the 13% of respondents who would never offer symmetrisation at the time of the initial surgery.

Hicks et al. studied the role of MRI in preoperative planning for patients undergoing therapeutic mammaplasty [13]. A third of women in this study (5/15) who underwent preoperative MRI were noted to have an additional area of malignant enhancement. The management of four of these patients (4/5) was altered to take into account these previously unrecognised lesions. This information was specifically used as part of the surgical planning for the TM to incorporate additional volume resection and enable breast reshaping. Despite no statistical difference between size on MRI and size on mammography versus final histological size, there was a greater correlation between size on MRI and final histological size. They concluded that MRI should be considered in selected patients. Our survey supported this suggestion as 80% of respondents would use MRI selectively, the common indications being lobular cancer, discrepancy in size on imaging, dense breasts, and neoadjuvant chemotherapy. Of interest, a recent meta-analysis found that the use of preoperative MRI in the staging of breast cancer was not associated with any improvement in local and distant recurrence [14].

Our study has shown a wide variation in how this procedure is coded by surgeons. Accurate coding and inclusion of comorbidities and complications are likely to affect reimbursement for this procedure, although assessment of this was beyond the scope of this study. Our own unpublished work has shown varying Healthcare Resource Group (HRG) codes allocated by coders resulting in a reimbursement discrepancy of up to 30% for similar matched patients. Currently, there is no dedicated HRG code for TM and ultimately reimbursement is dependent on coders' understanding of a complex hybrid oncological and plastic procedure, which is subject to interpretation. Proposals for the new OPCS 4.8 are currently being considered and efforts are underway to include TM.

One of the potential advantages of TM is to allow wide margins of excision, thus achieving lower rates of involved margins. A recent review found that rates of positive margin ranged from 0 to 36% with institutions reporting a 0% margin rate conducting intraoperative frozen section analysis [15]. No general consensus was achieved on involved margins with rates of reexcision ranging from 11 to 75% and completion mastectomy rates of 8–100%.

Only 20% of respondents from our survey would always offer mastectomy for involved margins. Reexcision can be challenging due to the glandular rearrangement during mammaplasty and should only be considered after careful discussion within the MDT if the operating surgeon is confident of the orientation of tumour bed and tumour histology is favourable. In addition, the original operating surgeon should undertake this procedure expeditiously to ensure accurate reexcision. We did not assess whether routine cavity shaves were performed at the time of operation and whether this has any bearing on rates of involved margins.

Oncoplastic breast conservation presents new challenges to the delivery of breast radiotherapy. The parenchymal rearrangement inherent to the majority of techniques may lead to the tumour bed being located at some distance from the skin incision and relocated out of the original tumour quadrant. In our practice, positive identification of the tumour bed is achieved with the use of metallic clips placed on the chest wall rather than the breast parenchyma. This ensures accurate planning for radiotherapy even where extensive glandular mobilisation has occurred.

Schaverien et al. have discussed the use of boost radiotherapy in oncoplastic breast conserving surgery in a systematic review [16]. The use of tumour bed boost radiotherapy was reported in only 11 studies out of 24, in 2 of which it was used for the treatment of incomplete margins only. Marking of the tumour bed was reported in 8 studies, all using metallic clips, with only one study reporting explicitly that the walls of the tumour cavity were clipped. They concluded that there is a need for better reporting of boost radiotherapy details in future studies of oncoplastic BCS to achieve more accurate radiotherapy planning. Oncoplastic MDMs have an important role in consolidating multidisciplinary working, allowing transparent decision making and standardisation of care [17]. They can be a useful platform for discussing the suitability of the patient for TM and/or planning the technique with plastic surgery and radiology colleagues. However, units may find it difficult to set up an additional MDM due to financial and time pressures. This may explain the low number of respondents who discussed patients in an oncoplastic MDM.

Tumour to breast ratio is an important factor in determining the suitability of patient for TM. A level 2 oncoplastic procedure for breast conserving procedures has been recommended for excision volumes of 20–50% [18]. It is interesting, however, that 30% of respondents did not consider a tumour to breast ratio of greater than 50% as an absolute contraindication. TM has extended the indications of BCS to very large and multifocal tumours. Although recent publications confirm the safety of this technique in obtaining clear surgical margins, the long term data on the oncological safety in large and multifocal tumours is limited [19]. In a review of 99 patients treated with TM, there was a significant correlation of incomplete excision rate with the tumour size and multifocality [20].

The negative effects of smoking on wound healing in patients undergoing breast reduction are well documented [21]. Breast cancer patients requiring a TM are a different subset and delaying surgery to allow for smoking cessation may not be realistic. Most surgeons in this survey would adopt a selective approach to smoking and only 20% considered this as an absolute contraindication to surgery. Where TM is considered in smokers, one option may be to avoid excessively thin flaps and plan a more conservative skin resections, thus reducing wound tension.

## 5. Conclusion

There is a wide variation in the practice of TM amongst OBS. Further research and guidance would be useful to standardise practice, particularly management of involved margins and coding for optimal reimbursement.
